# DRAM1 regulates autophagy and cell proliferation via inhibition of the phosphoinositide 3-kinase-Akt-mTOR-ribosomal protein S6 pathway

**DOI:** 10.1186/s12964-019-0341-7

**Published:** 2019-03-22

**Authors:** Ting Lu, Zhou Zhu, Junchao Wu, Hua She, Rong Han, Haidong Xu, Zheng-Hong Qin

**Affiliations:** 1 Department of Pharmacology and Laboratory of Aging and Nervous Diseases, Jiangsu Key Laboratory of Translational Research and Therapy for Neuro-Psycho-Diseases, College of Pharmaceutical Science, Soochow University, 199 Ren Ai Road, Suzhou, 215123 China; 2 Department of Pharmacology and Neurology, Emory University School of Medicine, Atlanta, GA USA

**Keywords:** DRAM1, Autophagy, Ribosomal protein S6, PI3K, Akt, IGF-1R, mTOR

## Abstract

**Background:**

Macroautophagy (hereafter autophagy) is a tightly regulated process that delivers cellular components to lysosomes for degradation. Damage-regulated autophagy modulator 1 (DRAM1) induces autophagy and is necessary for p53-mediated apoptosis. However, the signalling pathways regulated by DRAM1 are not fully understood.

**Methods:**

HEK293T cells were transfected with FLAG-DRAM1 plasmid. Autophagic proteins (LC3 and p62), phosphorylated p53 and the phosphorylated proteins of the class I PI3K-Akt-mTOR-ribosomal protein S6 (rpS6) signalling pathway were detected with Western blot analysis. Cellular distribution of DRAM1 was determined with immunostaining. DRAM1 was knocked down in HEK293T cells using siRNA oligos which is confirmed by quantitative RT-PCR. Cells were serum starved for 18 h after overexpression or knockdown of DRAM1 to decrease the rpS6 activity to the basal level, and then the cells were stimulated with insulin growth factor, epidermal growth factor or serum. rpS6 phosphorylation and rpS6 were detected with Western blotting. Similarly, after overexpression or knockdown of DRAM1, phosphorylation of IGF-1Rβ and IGF-1R were examined with Western blotting. Cell viability was determined with CCK-8 assay and colony formation assay. Finally, human cancer cells Hela, SW480, and HCT116 were transfected with the FLAG-DRAM1 plasmid and phosphorylated rpS6 and rpS6 were detected with Western blot analysis.

**Results:**

DRAM1 induced autophagy and inhibited rpS6 phosphorylation in an mTORC1-dependent manner in HEK293T cells. DRAM1 didn’t affect the phosphorylated and total levels of p53. Furthermore, DRAM1 inhibited the activation of the PI3K-Akt pathway stimulated with growth factors or serum. DRAM1 was localized at the plasma membrane and regulate the phosphorylation of IGF-1 receptor. DRAM1 decreased cell viability and colony numbers upon serum starvation. Additionally, DRAM1 inhibited rpS6 phosphorylation in several human cancer cells.

**Conclusions:**

Here we provided evidence that DRAM1 inhibited rpS6 phosphorylation in multiple cell types. DRAM1 inhibited the phosphorylation of Akt and the activation of Akt-rpS6 pathway stimulated with growth factors and serum. Furthermore, DRAM1 regulated the activation of IGF-1 receptor. Thus, our results identify that the class I PI3K-Akt-rpS6 pathway is regulated by DRAM1 and may provide new insight into the potential role of DRAM1 in human cancers.

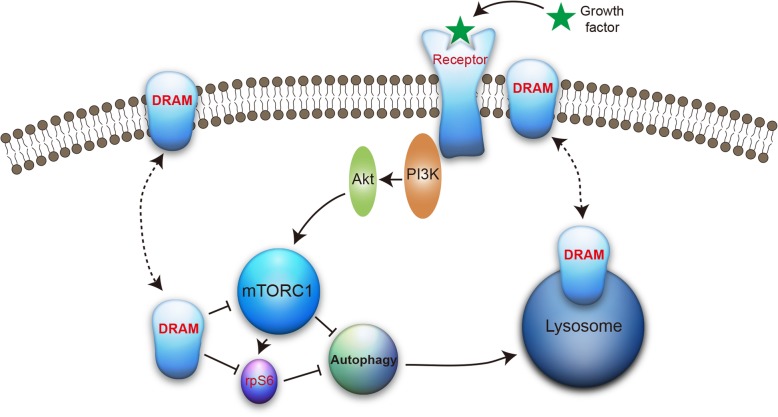

## Background

Macroautophagy (autophagy hereafter) is an evolutionarily conserved pathway through which intracellular proteins and organelles are delivered to lysosome for degradation [1]. During autophagy, isolated membrane or phagophore forms and encloses the cellular components to generate a double-membraned vesicle called autophagosome. Autophagosomes then fuse with other vesicles, such as endosomes, and finally fuse with lysosomes where the materials inside autophagosomes are degraded by lysosomal hydrolases for recycling [1, 2]. Autophagy can not only remove intracellular long-lived proteins or damaged organelles, but also can serve as an adaptive response upon various stresses such as nutrient starvation, infection, and protein aggregation [2]. Correspondingly, many cellular signalling pathways and molecules are activated to regulate different stages of autophagy. For example, PI3-Kinase-Akt-mTORC1, AMPK [3], p53 [4], Beclin 1 and Bcl-2 family proteins [5, 6] are all important autophagy regulators.

The role of PI3K-Akt-mTORC1 signalling in regulating autophagy and cell viability has been extensively studied. Growth factors bind and activate growth factor receptors, which lead to the phosphotyrosine residues of those receptors bind p110 subunit of PI3K and subsequently activate PI3K activity. For example, insulin stimulation activates insulin receptors involving the tyrosine autophosphorylation, which is necessary for kinase activation [7]. The activated PI3K enhances the production of phosphatidylinositol (3,4,5)-trisphosphate (PI(3,4,5)P_3_) on the plasma membrane, which recruits and binds to the pleckstrin-homology (PH) domains of downstream effectors, such as Akt (also known as protein kinase B, PKB) and PDK1. Through this binding, PDK1 phosphorylates Akt and stimulates its activity [8]. Akt regulates cell growth and cell survival by phosphorylating its downstream efforts such as mechanistic target of rapamycin complex 1 (mTORC1) [9, 10]. Several cellular processes including protein synthesis and autophagy are regulated by mTORC1. mTORC1 directly phosphorylates two key downstream effectors, eukaryotic translation initiation factor 4E-binding protein 1 (4EBP1) and p70S6 kinase 1 (p70S6K1), to promote protein synthesis and to inhibit autophagy [11].

Early studies by Meijer’s group showed that rapamycin, an inhibitor of mTOR, induced autophagy and inhibited the phosphorylation of ribosomal protein S6 in rat hepatocytes [12], which provides the first evidence that inhibition of autophagy and phosphorylation of rpS6 are under control of the same signalling pathway [12]. Ribosomal protein S6 is one of the components of the 40 S ribosomal subunit [13]. Previous study found that rpS6 underwent phosphorylation during rat liver regeneration [14]. In mammalian cells, the phosphorylation sites of rpS6 have been mapped to five clustered serine residues, Ser^235^, Ser^236^, Ser^240^, Ser^244^, and Ser^247^, which are located at the C-terminal domain of the protein [15]. Various signals, such as S6 kinase 1 (S6K1), p90 ribosomal S6 kinase (RSK) [16], protein kinase A (PKA) [17], and casein kinase 1 (CK1) [18], have been reported to regulate rpS6 phosphorylation. However, the physiological roles of rpS6 are still poorly understood [19].

Damage-regulated autophagy modulator 1 (DRAM1) was initially identified as the direct target gene of p53 during DNA damage [20]. DRAM1, an evolutionarily conserved protein, induces autophagy and is critical for p53-induced apoptosis [20]. Studies found that multiple isoforms of DRAM1 regulated autophagy process [20, 21]. Our previous study showed that DRAM1 regulated autophagic flux through enhancing lysosomal acidification [22]. However, the upstream signalling pathways that involved in DRAM1-induced autophagy are still unknown.

## Materials and methods

### Cell culture and reagents

HEK293T, Hela, HCT116 and SW480 cells were cultured at 37 °C in a humidified atmosphere of 95% air, 5% CO_2_ (v/v) in DMEM medium (GIBCO, Gaithesburger, MD, USA) supplemented with 10% (v/v) fetal bovine serum (GIBCO). Bafilomycin A1 was purchased from Enzo Life Sciences (Farmingdale, NY, USA), IGF-1 and EGF were obtained from Invitrogen (Burlingame, CA, USA), while LY294002, MK-2206 and LY2584702 were purchased from Selleck Chemicals (Houston, TX, USA).

### Constructs and transient transfections

The pCDNA3-DRAM1 plasmid was a kindly gift from Dr. Kevin Ryan (Beatson Institute for Cancer Research, Glasgow, UK). The 3 × FLAG expression plasmid was a kindly gift from Dr. Guanghui Wang (College of Pharmaceutical Science, Soochow University, Suzhou, China). FLAG-DRAM1 was constructed with these two vectors by PCR. Plasmids MYC-ULK1 and HA-Atg13 were purchased from Addgene. Transfections were performed using Lipofectamine 3000 (Invitrogen, Burlingame, CA, USA) following the manufacture’s protocol. Briefly, 1 μg plasmid (plus 1 μL P3000) and 2.5 μL Lipofectamine 3000 were separately mixed in 100 μL Opti-MEM (Invitrogen, Burlingame, CA, USA), and then mixed well and stood for 5 min before added to the cells. Cells were incubated with the liposome-DNA complexes for 24–48 h before harvest for experiments.

### Antibodies

Primary antibodies including anti-phospho-mTOR (Ser2448), anti-mTOR, anti-phospho-4EBP1 (Ser65), anti-4EBP1, anti-phospho-Akt (Ser473), anti-phospho-Akt (Thr308), anti-Akt, anti-phospho-rpS6 (Ser240/244), anti-phospho-rpS6 (Ser235/236), anti-rpS6, anti-p-p53 (Ser15), p53, anti-phospho-IGF-1Rβ (T1135/36/1150/51), IGF-1Rβ and anti-MYC antibodies were purchased from Cell Signaling Technology (Danfoss, MA, USA). The anti-rabbit IgG antibody was purchased from Jackson ImmunoResearch Laboratories (West Grove, PA, USA). Anti-S6K1 antibody was purchased from Abcam (Cambridge, UK). Anti-β-actin, anti-FLAG (M2) and anti-p62/SQSTM1 antibodies were purchased from Sigma (Saint Louis, MO, USA), anti-LC3 antibody was purchased from Medical & Biological Laboratories (Nagoya, Aichi, Japan). Secondary antibodies used in western blotting including IRDye 800CW Donkey anti-Mouse and anti-Rabbit IgG were purchased from Li-COR (Lincoln, NE, USA). Secondary antibodies used in immunofluorescence including AlexaFluor-555-conjugated goat anti-mouse and AlexaFluor-555-conjugated goat anti-rabbit antibodies were purchased from Invitrogen (Burlingame, CA, USA).

### siRNA knockdown and quantitative real-time RT-PCR

For transfection, HEK293T cells were plated in 6-well plate at 20–30% confluency, and siRNA duplexes (50 nM) were introduced into the cells using Lipofectamine RNAiMAX (Invitrogen, Burlingame, CA, USA) following the manufacturer’s instructions. The siRNA target sense sequences used were as follows: DRAM1 siRNA 1 (D1), 5′-AGCCACGAUGUAUACAAGATT-3′; and DRAM1 siRNA 2 (D2), 5′-CCACAGAAAUCAAUGGUGATT-3′; and negative control (Qiagen, Hilden, Germany), 5′-UUCUCUCCGAACGUGUCACGUTT-3′. The efficiency of knockdown was determined by qRT-PCR analysis 72 h after siRNA treatment. Briefly, total RNA was prepared with RNAiso Plus (Takara, Kusatsu, Shiga, Japan) and cDNA was synthesized from 1 μg of total RNA with PrimeScript RT-PCR Kit (Takara, Kusatsu, Shiga, Japan). qRT-PCR was performed using SYBR Premix Ex Taq II (Takara, Kusatsu, Shiga, Japan) with 7500 Real Time PCR System (Thermo Fisher Scientific, Rockford, IL, USA). The primers used were as follows: β-actin, 5′-CACCATTGGCAATGAGCGGTTC-3′ (forward) and 5′-AGGTCTTTGCGGATGTCCACGT-3′ (reverse); DRAM1: 5′-TCAAATATCACCATTGATTTCTGT-3′ (forward) and 5′-GCCACATACGGATGGTCATCTCTG-3′ (reverse).

### Cell proliferation assay

HEK 293 T and SW480 cells were plated at a density of 1 × 10^4^ cells per well in 100 μL DMEM medium supplemented with 10% FBS in 96-well plate for 24 h. After transfections, cells were cultured in 100 μL serum-containing or serum-free DMEM medium for 24 h. The proliferation of cells was evaluated using Cell Counting Kit-8 (Dojindo Laboratories, Kumamoto, Japan) assay. Briefly, 10 μL CCK-8 was added to each well of 96-well plates for 1-3 h at 37 °C and absorbance were measured at 450 nm using a microplate reader (ELX 800, Bio-Tek, Winooski, VT, USA).

### Cell colony formation assay

HEK293T or SW480 cells were transiently transfected with empty vector or FLAG-DRAM1 plasmid for 24 h and serum starved for another 24 h before the cells were counted and plated into 6-well plates, about 400 cells per well. Cells were cultured for about 2 weeks, then they were fixed with methanol and dyed using crystal violet. The plates were photographed, and the colonies were counted by a researcher who was unknown the experimental conditions.

### Western blot analysis

Cells were washed twice with PBS (pH 7.4) and harvested with ice-cold lysis buffer containing 40 mM Tris-HCl (pH 7.4), 150 mM NaCl, 0.5% sodium deoxycholate, 1% NP40 and EDTA-free complete protease inhibitor and Phospho-Stop inhibitor (Roche, Basel, Switzerland). Cell lysates were centrifuged at 11,000 rcf for 15 min at 4 °C. The supernatants were collected and protein concentrations were determined using the BCA protein assay kit (Takara, Kusatsu, Shiga, Japan). Equal amounts of protein samples were separated by sodium dodecyl sulfate-polyacrylamide gel electrophoresis (SDS-PAGE) and transferred to nitrocellulose membranes (Millipore, Billerica MA, USA). After washed with TBST buffer for 5 min twice, the membranes were blocked with 5% non-fat milk in TBST buffer for 1 h at room temperature and then incubated with primary antibodies overnight at 4 °C as follows: anti-LC3 (1:1000), anti-p62 (1:2000), anti-p-ERK (1:1000), anti-ERK (1:1000), anti-p-mTOR (1:1000), anti-mTOR (1:1000), anti-p-Akt (1:1000), anti-Akt (1:1000), anti-p-p70S6K1 (1:1000), anti-p70S6K1 (1:1000), anti-p-rpS6 (1:1000), anti-S6 (1:1000), or β-actin (1:10000) overnight at 4 °C. Then the membranes were washed with TBST and incubated with Li-COR secondary antibody (donkey anti-rabbit or mouse, 1:20000) for 1 h at room temperature. Immuno-signals was detected using the Odyssey Western Blot Analysis system (Li-COR Biosciences, Lincoln, NE, USA). The signal intensities of protein bands were quantitatively analyzed with Image J software and normalized to the loading control β-actin.

### Immunofluorescence

Cover glasses pretreated with 75% ethanol were plated into 24-well plate, and cells were seeded at a density of 2–5 × 10^4^ cells per well. After treatments, cells were washed twice with PBS, fixed with 4% paraformaldehyde for 20 min at room temperature, and then washed three times with PBS, permeabilized with 0.2% Triton X-100 in PBS for 15 min at 4 °C and washed three times with PBS. Then the cells were blocked with 2% goat serum for 1 h at room temperature and incubated with mouse anti-FLAG (1:1000) overnight at 4 °C. On the next day, cells were stained with the appropriate fluorescence-conjugated secondary antibodies and mounted on glass slides in aqueous mounting medium (Sigma, Saint Louis, MO, USA). All slides were imaged with laser scanning confocal microscopy (Zeiss LSM 710, Carl Zeiss, Jena, Germany).

### Cell surface biotinylation

Plasma membrane proteins of HEK293T cells were surface biotinylated by incubation of ~ 1 × 10^7^ cells/ml in membrane-impermeable biotinylation reagent Sulfo-NHS-Biotin (1 mg/ml, in HBSS; Abcam, Cambridge, UK) for 30 min following the manufacturer’s protocol. After that, free biotin was quenched by washing cells twice with 50 mM glycine in HBSS. And then they were lysed in cell lysis buffer. Biotin-labeled proteins were isolated using streptavidin-agarose beads (Thermo Fisher Scientific, Rockford, IL, USA) and detected by immunoblotting with indicated antibodies following SDS-PAGE.

### Statistical analysis

Data were expressed as mean ± standard error of the mean (SEM). Statistical analysis was carried out using GraphPad Prism (GraphPad Software, San Diego, CA), either by one-way ANOVA followed by Dunnett’s t-test for multiples means or by two-tailed unpaired t-test for two means. Differences were considered significant when *P* < 0.05.

## Results

### DRAM1 increases autophagosome formation and inhibits the phosphorylation of rpS6

**Fig. 1:**
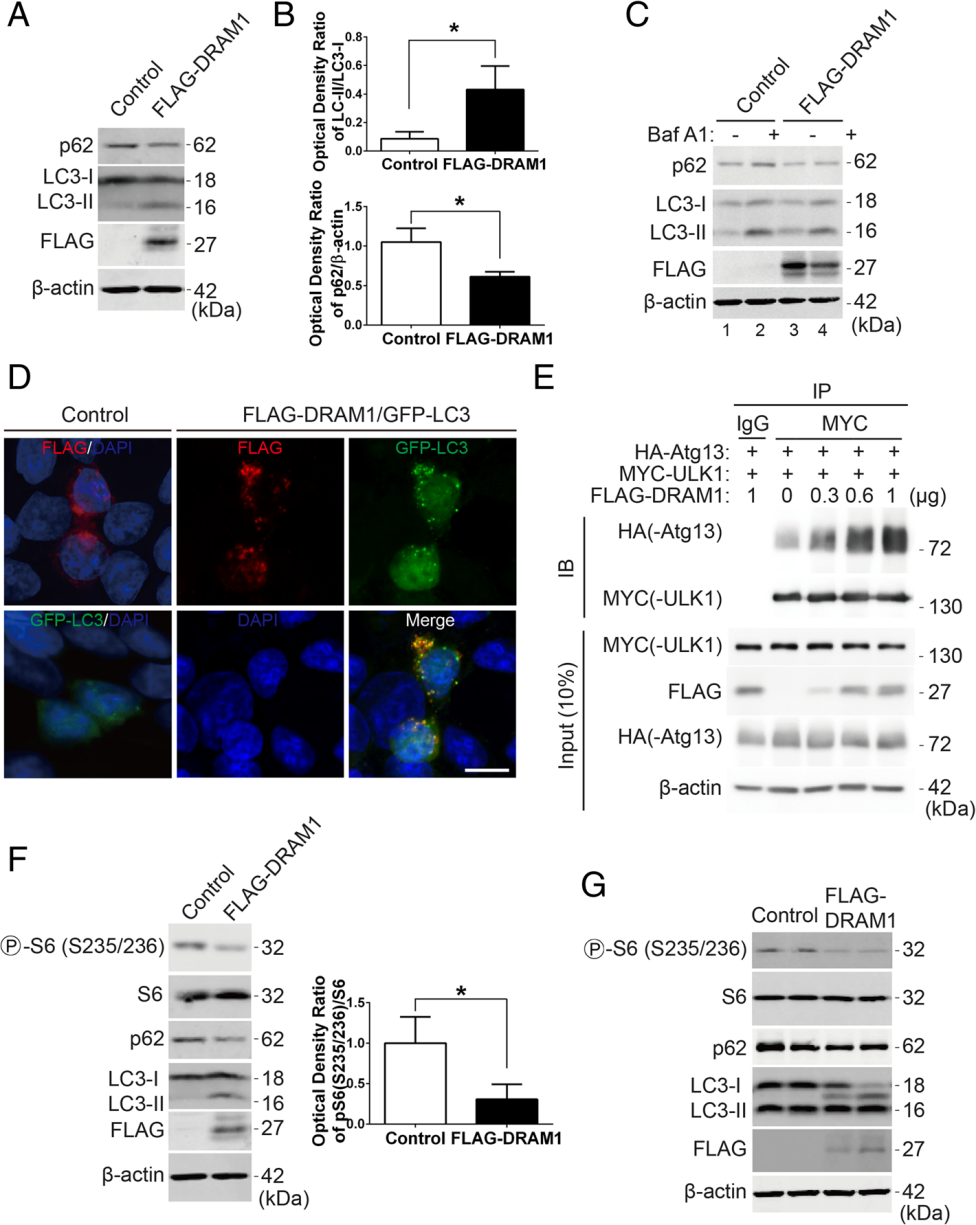
DRAM1 enhances autophagosome formation and downregulation of rpS6 phosphorylation. **a** HEK293T cells were transfected with FLAG empty vector or FLAG-DRAM1 for 24 h, the protein levels of p62, LC3, FLAG and β-actin were detected with Western blot analysis. **b** Quantitative analysis of the optical densities of LC3-II and p62 in HEK293T cells transfected with FLAG-DRAM1. Data represent mean ± SEM for combined data from three independent experiments. **c** HEK293T cells were transfected with FLAG empty vector or FLAG-DRAM1 for 48 h and then were treated with DMSO or Bafilomycin A1 (100 nM) for 6 h before harvesting. **d** Representative confocal images of HEK293T cells transfected with FLAG-DRAM1 or GFP-LC3 alone in control group and co-transfected with FLAG-DRAM1 and GFP-LC3. Scale bar represents 10 μM. **e** HEK293T cells were transfected with HA-Atg13, MYC-ULK1 and different amount of FLAG-DRAM1 for 24 h. The whole cell lysates (200 μg) were immunoprecipitated with an anti-IgG or anti-MYC antibody, and the precipitates were detected with an anti-HA antibody. **f** HEK293T cells were transfected with FLAG empty vector or FLAG-DRAM1 for 24 h, and the protein levels of p-rpS6 (S235/236), rpS6, p62, LC3, FLAG and β-actin were detected with Western blot analysis. Levels of p-rpS6 (S235/236) and rpS6 were quantitatively analyzed. Data represent mean ± SEM for combined data from three independent experiments. **g** Hela cells were transfected with FLAG empty vector or FLAG-DRAM1 for 36 h, and the protein levels of p-rpS6 (S235/236), rpS6, p62, LC3, FLAG and β-actin were detected with Western blot analysis. *p < 0.05 vs control

The previous study reported that DRAM1 enhanced autophagy activity [20]. Firstly, we sought to confirm if DRAM1 induced autophagy under our experimental condition. As there is no reliable antibodies for detection of DRAM1 in cultured cells, we constructed 3 × FLAG-tagged DRAM1 plasmid. We transfected FLAG-DRAM1 plasmid into HEK293T cells and examined the autophagy marker LC3 and autophagy substrate p62/SQSTM1. Overexpression of DRAM1 induced the turnover of LC3-I into LC3-II and decreased the protein level of p62 (Fig. 1a and b). As expected, blockage of autophagic flux with Bafilomycin A1 (Baf A1), a specific inhibitor of vacuolar H^+^ ATPase, dramatically accumulated both LC3-II and p62 in control group (Fig. 1c, lane 2). Expression of DRAM1 increased LC3-II and decreased p62 protein level under basal condition (Fig. 1c, lane 3). Interestingly, BafA1 induced accumulation of levels of p62 and LC3-II was blunted by overexpression of DRAM1 (Fig. 1c, lane 4 v. s. lane 2). When co-transfected GFP-LC3 and FLAG-DRAM1 plasmids into HEK293T cells, GFP-LC3 dots significantly increased in the presence of DRAM1 compared with GFP-LC3 alone in control group, indicating the enhanced formation of autophagosomes (Fig. 1d). DRAM1 distributed in punctate and diffusion patterns when expressed alone and was partially co-localized with GFP-LC3 (Fig. 1d) when co-expressed, which was consistent with the previous report [21]. Previous study showed that ULK1 and Atg13 forms a stable complex that is essential for autophagosome formation [23]. A recent study showed that p38 MAPK inhibited autophagy by phosphorylating ULK1 and prevented it from binding to the downstream effector Atg13 upon inflammatory stimulation [24]. To explore whether DRAM1 affects ULK1-Atg13 complex, we transfected HEK293T cells with ULK1, Atg13 and DRAM1, immunoprecipitated ULK1 and analyzed Atg13-ULK1 binding in the presence of different expression levels of DRAM1. Data showed that DRAM1 could enhance ULK1-Atg13 binding in a dose-dependent manner (Fig. 1e), which was consistent with the data that DRAM1 increased autophagosome formation in GFP-LC3 assay. Thus, our data showed that DRAM1 induced autophagy and increased autophagosome formation in HEK293T cells.

To investigate the signalling pathways involved in DRAM1-induced autophagy, we overexpressed DRAM1 and examined the signalling pathways regulating autophagy. As mentioned before, phosphorylation of ribosomal protein S6 (rpS6) exerts inhibitory role on autophagic proteolysis [12]. We found that overexpression of DRAM1 dramatically inhibited rpS6 phosphorylation at Ser^235/236^ upon autophagy induction in HEK 293 T cells (Fig. 1f). In Hela cells, overexpression of DRAM1 induced the turnover of LC3-I into LC3-II and decreased the protein level of p62, indicating that DRAM1 induces autophagy in these cells. Notably, under this condition, DRAM1 also inhibited the phosphorylation of rpS6 at Ser^235/236^ (Fig. 1g). These data suggested that DRAM1-induced autophagy was accompanied by the inhibition of rpS6 phosphorylation.

### DRAM1 inhibits rpS6 phosphorylation via the mTORC1-p70S6K signalling pathway

**Fig. 2:**
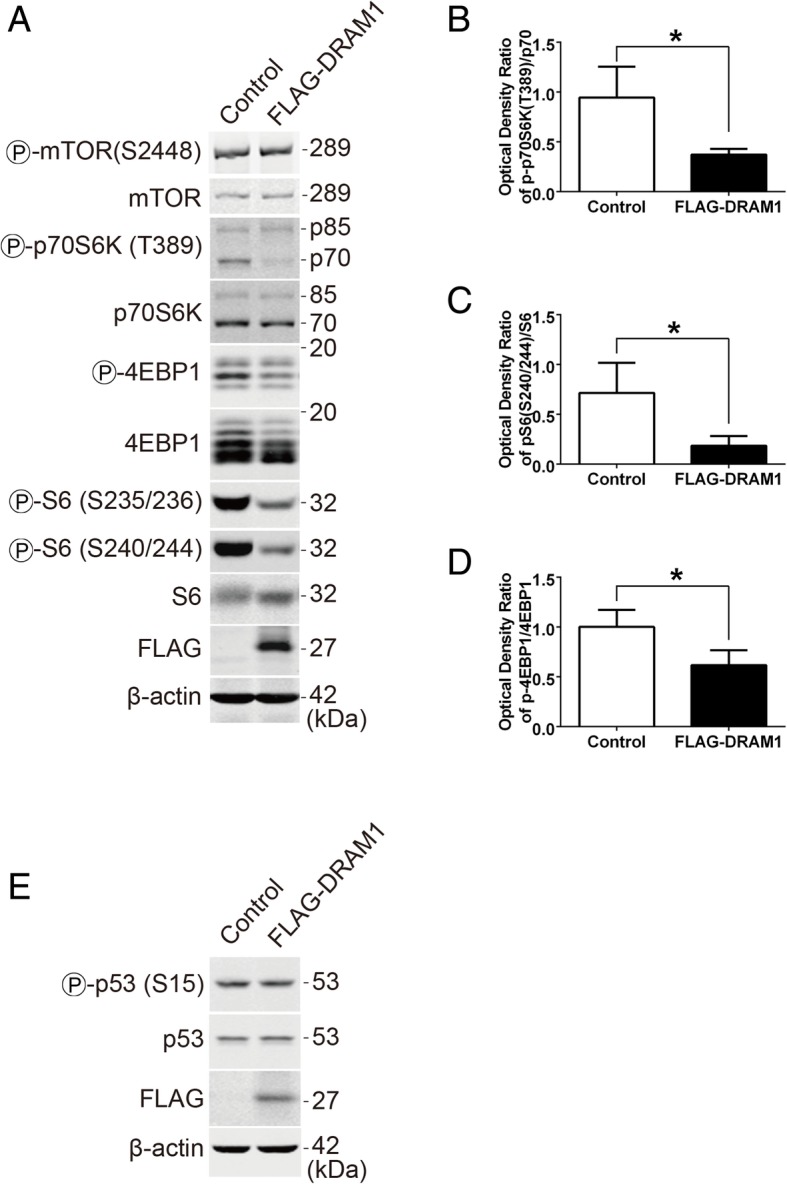
DRAM1 inhibits the mTORC1 signalling. **a** HEK293T cells were transfected with FLAG empty vector or FLAG-DRAM1 for 48 h, and the protein levels of p-mTOR (S2448), mTOR, p-p70S6K (T389), p70S6K, p-4EBP1, 4EBP1, p-rpS6 (S235/236, S240/244), rpS6, FLAG and β-actin were detected with immunoblotting. **b**, **c** and **d** Quantitative analysis of p-rpS6 (S240/244), p-p70S6K and p-4EBP1 in HEK293T cells transfected with FLAG-DRAM1. Data represent mean ± SEM of combined data from three independent experiments. **e** HEK293T cells were transfected with FLAG empty vector or FLAG-DRAM1 for 48 h and the protein levels of p-p53 (S15), p53, FLAG and β-actin were detected with immunoblotting. *p < 0.05 vs control

To investigate the potential mechanisms involved in the inhibition of rpS6 phosphorylation by DRAM1, we analyzed the mTORC1-p70S6K pathway. DRAM1 overexpression reduced the phosphorylation of p70 but not p85 (Fig. 2a and b). DRAM1 also dramatically downregulated the phosphorylation of both rpS6 at Ser^240/244^ (Fig. 2a and c) and 4EBP1 (Fig. 2a and d), suggesting that the activity of p70S6K was decreased, though the phosphorylation of mTOR itself had no change (Fig. 2a). In addition, overexpression of FLAG-DRAM1 didn’t affect the phosphorylation of p53 at Ser^15^ as well as the total level of p53 (Fig. 2e), indicating that the effects of DRAM1 overexpression on rpS6 might be independent of p53 activity under our experimental condition. Overall, these data indicated that DRAM1 might inhibit rpS6 phosphorylation through mTORC1-p70S6K pathway.

### DRAM1 inhibits the activation of the PI3K-Akt pathway stimulated by growth factors and serum

**Fig. 3:**
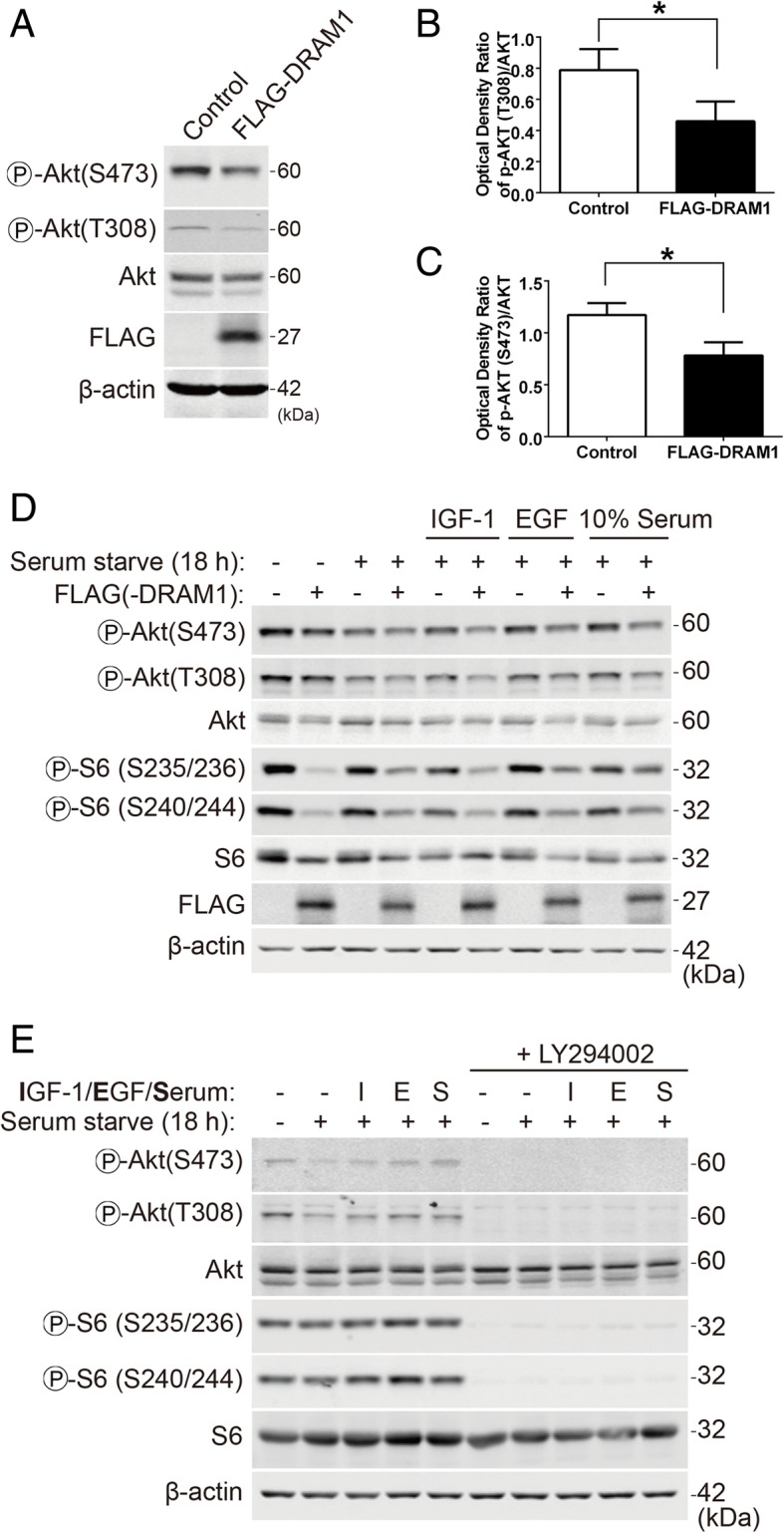
DRAM1 inhibits the activation of the PI3K-Akt pathway stimulated with growth factors and serum. **a** HEK293T cells were transfected with FLAG empty vector or FLAG-DRAM1 for 24 h and the protein levels of p-Akt (S473 and T308), FLAG and β-actin were detected with immunoblotting. **b** and **c** Quantitative analysis of the optical densities of p-Akt (T308, S473) in HEK293T cells transfected with FLAG-DRAM1. Data represent mean ± SEM of combined data from three independent experiments. **d** HEK293T cells were transfected with FLAG empty vector or FLAG-DRAM1 for 24 h, and then starved for another 18 h before stimulating with IGF-1 (5 ng/ml), EGF (50 ng/ml) and serum (10%) for 10 min. The protein levels of p-Akt (S473 and T308), Akt, p-rpS6 (S235/236, S240/244), rpS6, FLAG and β-actin were detected with immunoblotting. **e** HEK293T cells were transfected with FLAG empty vector or FLAG-DRAM1 for 24 h, and then starved for another 18 h before stimulated with IGF-1, EGF or serum (10%) for 10 min in the presence of or absence of LY294002 (50 μM). The protein levels of p-Akt (S473 and T308), Akt, p-rpS6 (S235/236 and S240/244), rpS6 and β-actin were detected by immunoblotting. *p < 0.05 vs control

The above data suggested that DRAM1 might play a role upstream of mTORC1 to regulate the phosphorylation of rpS6. To investigate the how mTOR is regulated by DRAM1, we examined the class I PI3K-Akt pathway. As shown in Fig. 3a, overexpression of DRAM1 inhibited the phosphorylation of Akt at Thr^308^ and Ser^473^ (Fig. 3a, b and c). To further confirm whether DRAM1 could inhibit the activation of the class I PI3K-Akt pathway, cells were transfected with DRAM1 and serum starved for another 18 h before stimulated with growth factors or serum. The rationale to do so is that long-term starvation decreases the rpS6 kinase activity to the lowest basal levels [16], which renders the cells more sensitive to stimulations than the normal condition and thus makes the stimulated effects of growth factors or serum easier to observe. After transfected and starved, the cells were stimulated with insulin-like growth factor 1 (IGF-1), epidermal growth factor (EGF) and serum. IGF-1, EGF and serum stimulated the phosphorylation of Akt and rpS6, while DRAM1 dramatically inhibited the phosphorylation of Akt at Thr^308^ and Ser^473^ and rpS6 at Ser^235/236^ and Ser^240/244^ (Fig. 3d). To further confirm that the PI3K-Akt pathway indeed regulates rpS6 phosphorylation in HEK293T cells, we applied the class I PI3K inhibitor LY294002 and analyzed rpS6 phosphorylation. As shown in Fig. 3e, LY294002 treatment totally abolished the phosphorylation of Akt and rpS6 at both sites. Furthermore, IGF-1, EGF and serum induced phosphorylation of Akt and rpS6 were also blocked by LY294002, indicating that rpS6 phosphorylation is under the regulation of PI3K-Akt signalling pathway in HEK293T cells. These results indicated that DRAM1 could inhibit the phosphorylation and activation of Akt stimulated by growth factors.

**Fig. 4:**
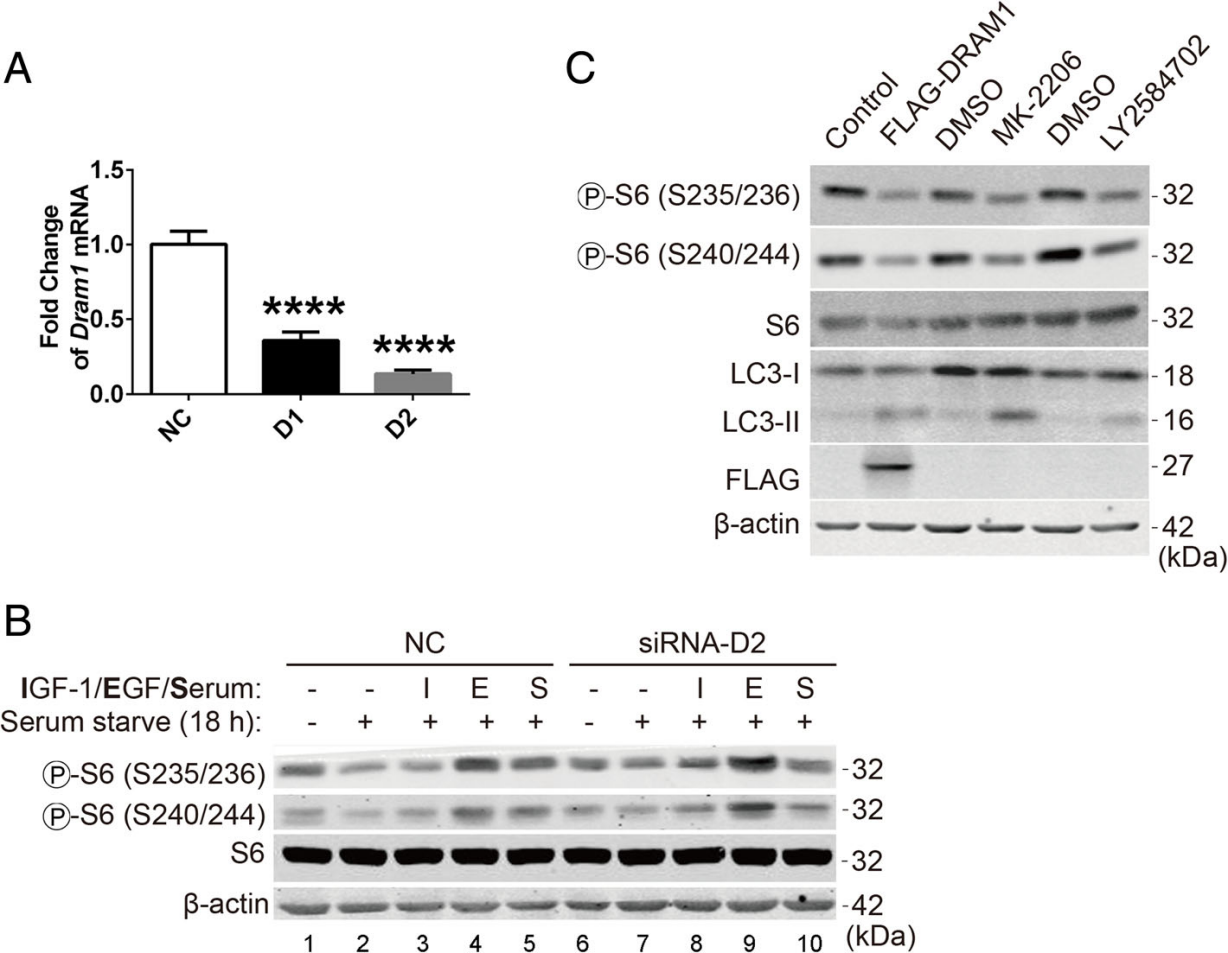
DRAM1 suppresses growth factors stimulated rpS6 phosphorylation. **a** HEK293T cells were transfected with oligos of negative control (NC), DRAM1 siRNA 1 (D1) or DRAM1 siRNA 2 (D2) with the final concentration of 50 nM for 48 h. The mRNA levels were detected with qRT-PCR and normalized to β-actin. Data represent mean ± SEM of combined data from three independent experiments. **b** HEK293T cells were transfected with negative control or DRAM1 siRNA for 24 h, and then starved for another 18 h before stimulated with IGF-1 (5 ng/ml), EGF (50 ng/ml) or serum (10%) for 10 min. The protein levels of p-rpS6 (S235/236, S240/244), rpS6 and β-actin were detected with immunoblotting. **c** HEK293T cells were transfected with FLAG empty vector or FLAG-DRAM1 plasmids for 24 h, or the cells were treated with DMSO, MK-2206 (5 μM) or LY2584702 (10 μM) for 24 h, respectively. The protein levels of p-rpS6 (S235/236, S240/244), rpS6, LC3, FLAG and β-actin were detected with immunoblotting. ****p < 0.001 vs the NC group

To test whether downregulation of DRAM1 affects the activation of the class I PI3K-Akt pathway, we knocked down endogenous DRAM1 with siRNA oligos. As shown in Fig. 4a, two siRNA oligos dramatically downregulated the mRNA level of DRAM1 with a better effect for the second oligo (D2), so we chose D2 for further experiments. DRAM1 was knocked down for 24 h and then cells were starved for another 18 h followed by stimulating with IGF-1, EGF or serum (Fig. 4b). In negative control (NC) group, serum starvation reduced the phosphorylation level of rpS6 to a low basal level. Consistent with previous data, stimulation with EGF, IGF-1 or serum enhanced the phosphorylation levels of rpS6 at both sites in HEK293T cells. (Fig. 4b, lanes 1–5). DRAM1 knockdown itself doesn’t affect the basal phosphorylation levels of rpS6 in normal (Fig. 4b, lanes 1,2) and starved conditions (Fig. 4b, lanes 6,7). Though stimulated the cells with serum didn’t induce a dramatical change of rpS6 phosphorylation between these two groups, stimulation with IGF-1 or EGF could further increased the phosphorylation levels of rpS6 at both sites comparing with the NC group (Fig. 4b, lanes 3 vs 8 and lanes 4 vs 9), indicating that DRAM1 knockdown cause the overactivation of rpS6 phosphorylation stimulated with growth factors, probably via the PI3K-Akt pathway.

In addition, we used the inhibitors of Akt (MK-2206) and p70S6K (LY2584702) to inhibit the activation of Akt and p70S6K, respectively. The results showed that both MK-2206 and LY2584702 could mimic the inhibitory effect of DRAM1 on rpS6 phosphorylation and autophagy induction (Fig. 4c). Overall, these data indicate that DRAM1 inhibits the activation of the class I PI3K-Akt-mTORC1-rpS6 signalling cascade stimulated by growth factors and serum in HEK293T cells.

### DRAM1 localizes in plasma membrane and regulates the activation of IGF-1 receptors

**Fig. 5:**
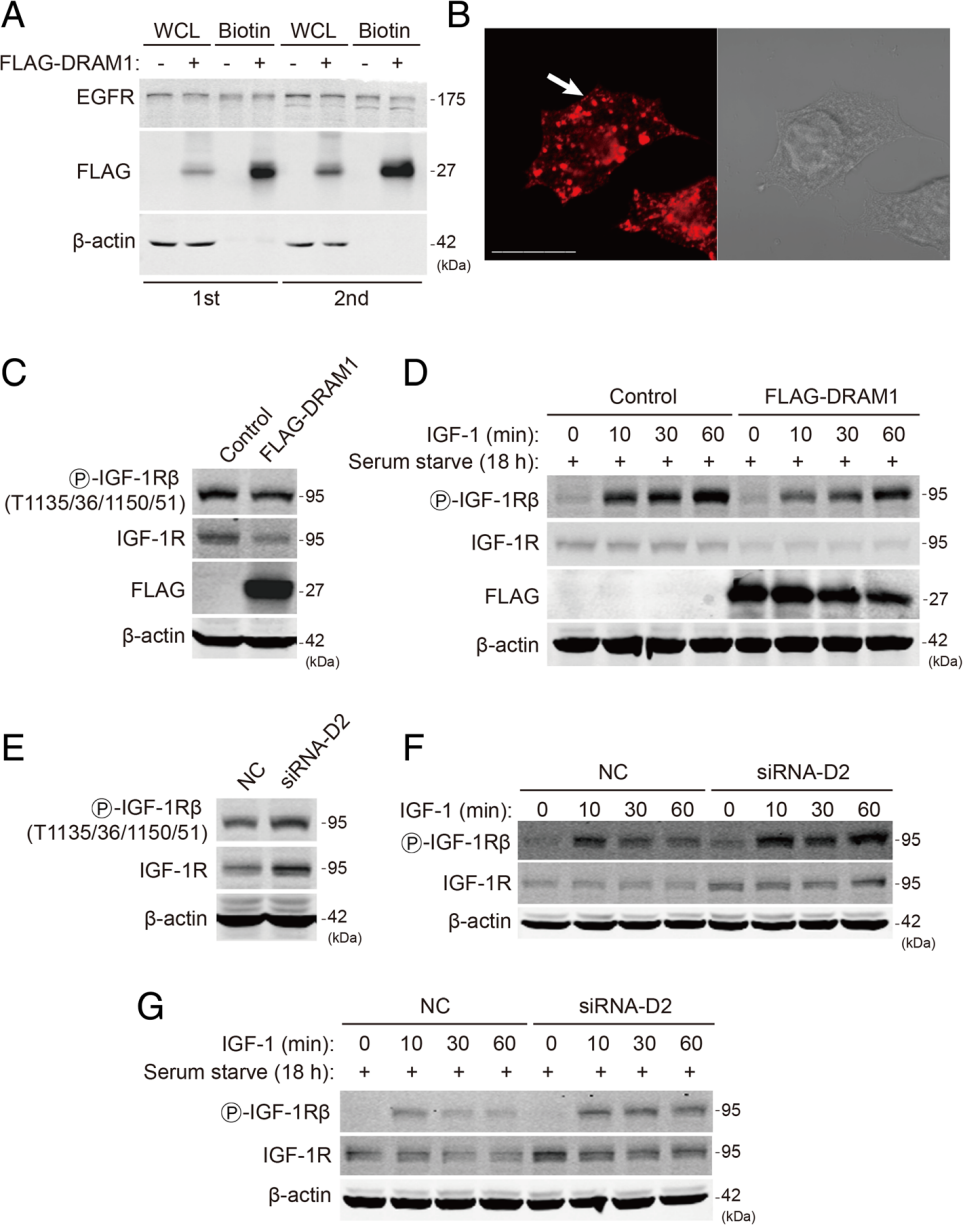
DRAM1 localizes in plasma membrane and regulates the activation of IGF-1 receptors. **a** Surface of HEK293T cells was biotinylated. The protein levels of EGFR, FLAG and β-actin in whole cell lysate (WCL) and Biotin fractions were detected with immunoblotting. The data showed two independent experiments (1st and 2nd). **b** HEK293T cells were transfected with FLAG-DRAM1 plasmid for 24 h, the cells were fixed and stained with FLAG antibody (red). Representative immunofluorescent and phase contrast pictures were shown. Arrow indicated the FLAG staining on the membrane. Scale bar represents 10 μm. **c** and **d** HEK293T cells were transfected with FLAG empty vector or FLAG-DRAM1 for 36 h (**c**) or starved for another 18 h (**d**) before stimulated with IGF-1 (5 ng/ml) for the indicated time. The protein levels of p-IGF-1R β (T1135/36/1150/51), IGF-1R, FLAG and β-actin were detected by immunoblotting. **e** and **f** HEK293T cells were transfected with negative control or DRAM1 siRNA for 48 h (**e**) or stimulated with IGF-1 (5 ng/ml) for the indicated time (**f**). **g** HEK293T cells were transfected with negative control or DRAM1 siRNA for 48 h and starved for another 18 h (**g**) before stimulated with IGF-1 (5 ng/ml) for the indicated time. The protein levels of p-IGF-1R β (T1135/36/1150/51), IGF-1R and β-actin were detected with immunoblotting

Given that DRAM1 processes six hydrophobic transmembrane domains [20] and previous study showed that DRAM1 was partially localized in the plasma membrane [25], it is possible that DRAM1 regulates signalling cascades on cell membrane. To test whether FLAG-DRAM1 could localized in plasma membrane in our system, we used membrane-impermeable biotin to label membrane proteins. As shown in Fig. 5a, strong FLAG signal could be detected in biotin-labeled proteins. Immunostaining results also showed that FLAG-DRAM1 diffused in the cytosol in larger granular structures, and signals could also be found in the membrane or near the membrane, as indicated by the arrow (Fig. 5b). As DRAM1 inhibited IGF-1-stimulated activation of class I PI3K pathway, we speculated that DRAM1 may affect the receptor of insulin-like growth factor in the membrane. Overexpression of DRAM1 decreased the protein level of IGF-1 receptor (IGF-1R) β with slightly downregulation of the phosphorylation of IGF-1R β (Fig. 5c). When cells were starved before stimulated with IGF-1, DRAM1 overexpression dramatically decreased the phosphorylation of IGF-1R β (Fig. 5d). Consistently, knockdown of DRAM1 for 48 h dramatically increased the phosphorylation and total levels of IGF-1R (Fig. 5e). In the presence of IGF stimulation, knockdown of DRAM1 led to a robust phosphorylation of IGF-1R in different time course compared with the negative control group (Fig. 5f). Similar results could be obtained in DRAM1-knockdown cells serum-starved for another 18 h before stimulated with IGF (Fig. 5g). These results demonstrated that DRAM1 localized in the plasma membrane and regulated the activation of IGF-1R upon IGF stimulation.

### DRAM1 modulates the kinetics of rpS6 phosphorylation and reduces cell proliferation under serum starvation condition

**Fig. 6:**
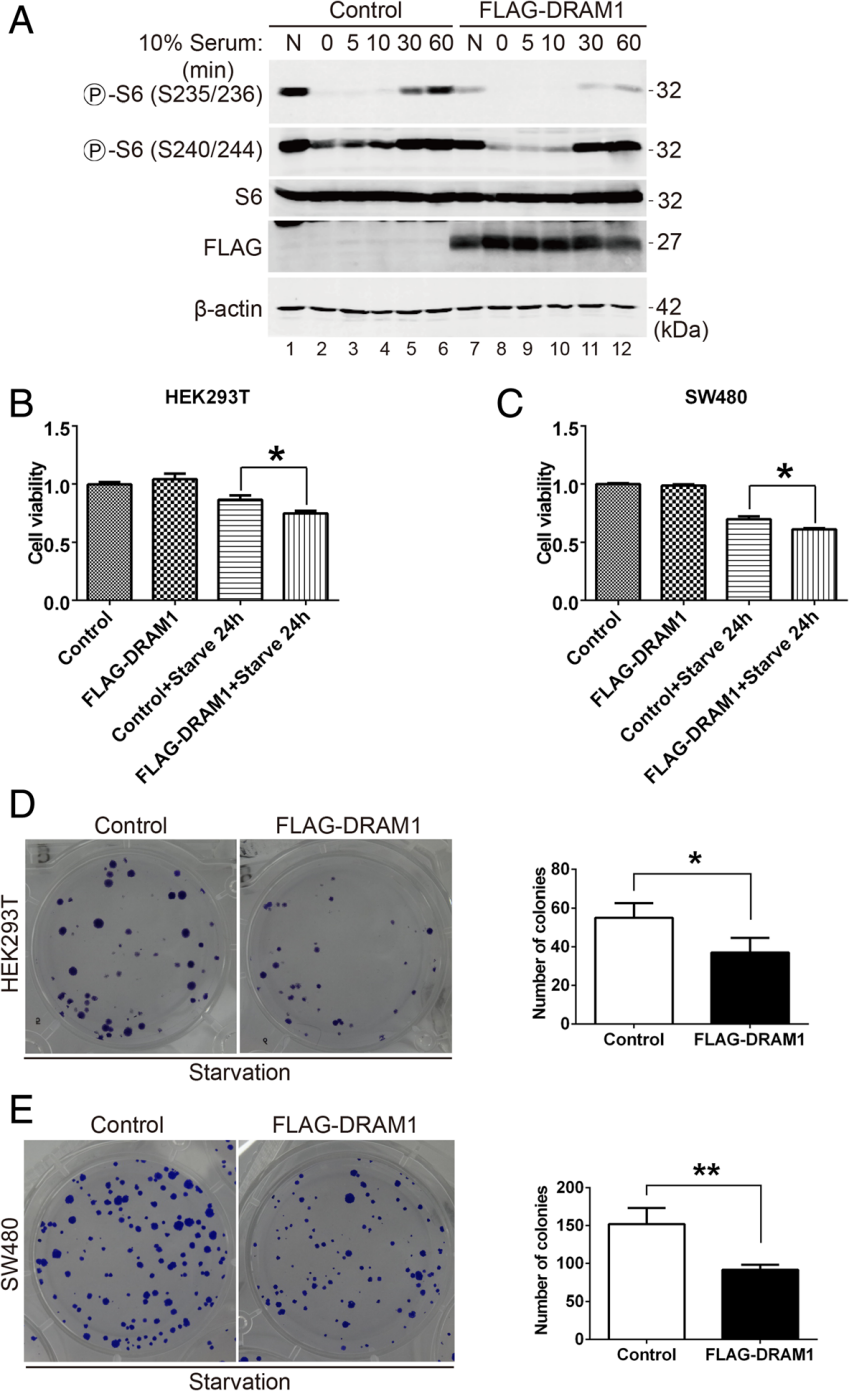
DRAM1 inhibits serum-stimulated phosphorylation of rpS6 and reduces cell proliferation under serum starvation condition. **a** HEK293T cells were transfected with FLAG empty vector or FLAG-DRAM1 for 24 h followed by starvation for another 18 h before stimulating with serum (10%) over a time-course. Both phosphorylation sites of rpS6 (S235/236 and S240/244) were detected with phospho-specific antibodies. Total protein levels of rpS6, FLAG and β-actin were also detected with immunoblotting. **b** and **c** HEK293T and SW480 cells were transfected with FLAG empty vector or FLAG-DRAM1 plasmids for 24 h, and the cells were starved for another 24 h before examining cell viability using CCK-8 kit. **d** and **e** HEK293T cells were transfected with FLAG vector or FLAG-DRAM1 for 24 h, and serum starved for another 24 h, then the cells were counted and seeded onto 6-well plate. Two weeks late, the colony number was counted. *p < 0.05 and **p < 0.01 vs the indicated groups

To characterize the kinetics of rpS6 phosphorylation, HEK293T cells were firstly starved and then stimulated with serum over a time-course (Fig. 6a). Data showed that rpS6 was phosphorylated at all four sites (Ser^235/236^, Ser^240/244^) analyzed and reached the peak level at 30–60 min of stimulation (Fig. 6a, lanes 1–6). However, DRAM1 dramatically blunted rpS6 phosphorylation kinetics at both Ser^235/236^ and Ser^240/244^ under serum stimulation (Fig. 6a, lanes 7–12). The kinetics of rpS6 phosphorylation inhibited by DRAM1 were slightly different between these sites. DRAM1 exerted more potent inhibitory effect on the phosphorylation of rpS6 at Ser^235/236^ than that at Ser^240/244^. Meanwhile, phosphorylation of Ser^240/244^ reached much higher levels and occurred with much faster kinetics than Ser^235/236^, suggesting that phosphorylation of these sites might be regulated by different signalling pathways. These data also showed that DRAM1 not only inhibited rpS6 phosphorylation at different sites, but also modulated the kinetics of rpS6 phosphorylation under serum stimulation. Moreover, though overexpression of DRAM1 alone did not reduce cell viability as previous reported, DRAM1 reduced cell viability under serum starvation conditions in HEK293T cell as well as in SW480 (Fig. 6b and c). To determine the long-term effects of DRAM1 on cell viability, we performed colony formation assay. As shown in Fig. 6d and e, when cells were transfected with plasmids and starved, the ability of colony formation of FALG-DRAM1-expressed cells were dramatically decreased comparing to that of control cells. These data suggest that DRAM1 may sensitize cells to lose viability under nutrient stress conditions, which is consistent with the observations that DRAM1 inhibits the class I PI3K-Akt-mTORC1-rpS6 pathway.

### DRAM1 inhibits rpS6 phosphorylation in human cancer cells

**Fig. 7:**
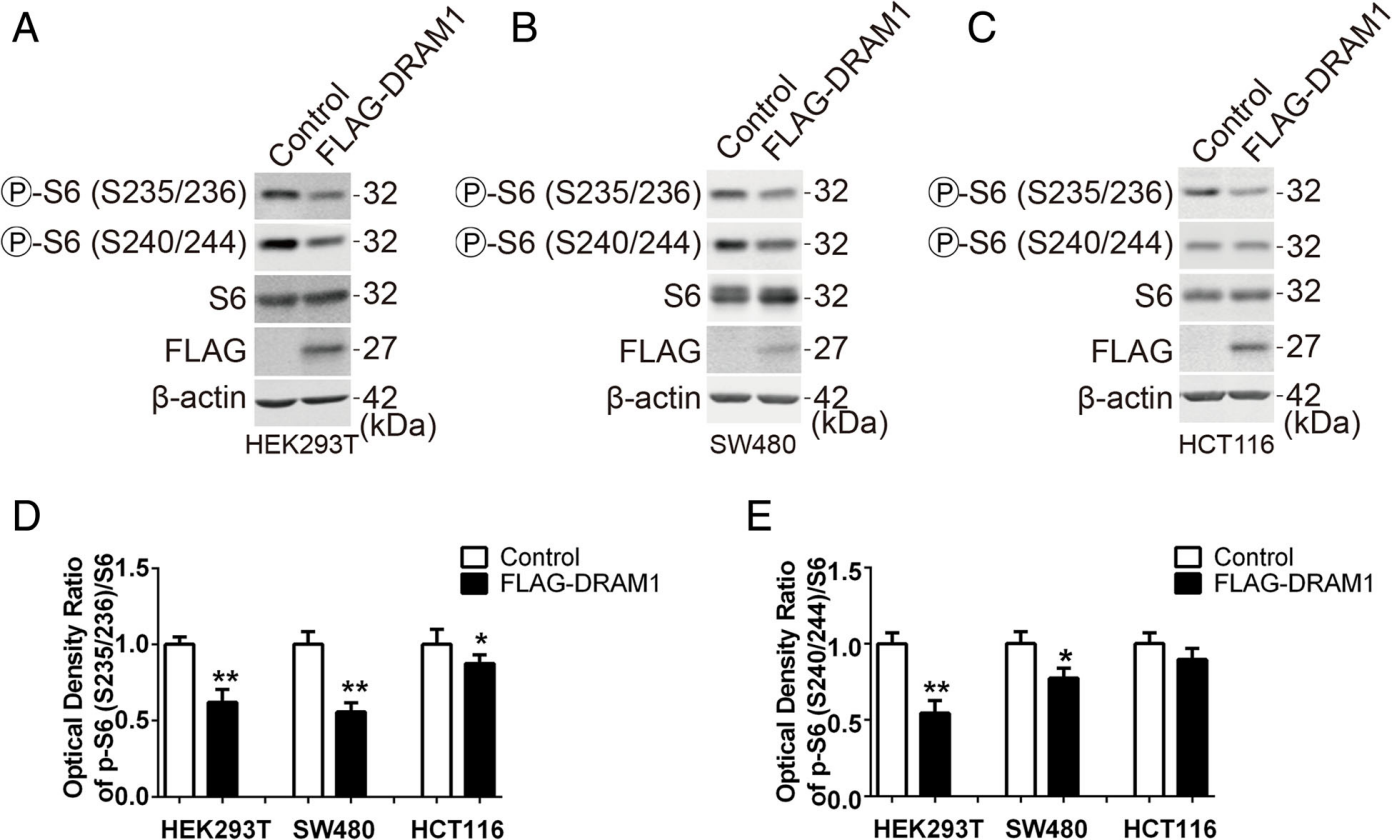
DRAM1 inhibits rpS6 phosphorylation in human cancer cells. **a**, **b** and **c** HEK293T, SW480 and HCT116 cells were transfected with FLAG empty vector or FLAG-DRAM1 plasmids for 24 h. The protein levels of p-rpS6 (S235/236, S240/244), rpS6, FLAG and β-actin were detected with immunoblotting. **d** and **e** Quantitative analysis of the optical densities of p-rpS6 (S235/236, S240/244) and rpS6. Data represent mean ± SEM of combined data from three independent experiments. *p < 0.05 and **p < 0.01 vs the indicated groups

The previous study identified DRAM1 as a potential tumor-suppressor in human cancer [20]. To investigate whether DRAM1 could inhibit the phosphorylation of rpS6 in human cancer cell lines, we overexpressed DRAM1 in human cancer cells. Using HEK293T cells as a positive control (Fig. 7a), we found that DRAM1 inhibited rpS6 phosphorylation in human colon cancer cells, SW480 (Fig. 7b) and HCT116 (Fig. 7c), with phosphorylation at Ser^235/236^ more significantly affected by DRAM1 than the site at Ser^240/244^ (Fig. 7d and e). These data demonstrated that DRAM1 inhibited rpS6 phosphorylation in human colon cancer cells.

## Discussion

DRAM1 has been identified as the direct p53 target gene more than a decade ago [20, 25]. Initial study showed that DRAM1 induced autophagy and was necessary for p53-induced apoptosis [20]. However, the signalling pathways involved in DRAM1-induced autophagy and apoptosis are still not clear. In this study, we demonstrated that DRAM1 inhibited the phosphorylation of rpS6 in multiple cell lines. Furthermore, DRAM1 inhibited the activation of the class I PI3K-Akt pathway stimulated with growth factors and serum. Our results suggest that the class I PI3K-Akt-mTORC1-rpS6 pathway plays a key role in DRAM1-induced autophagy and apoptosis.

Early study found that DRAM1-inducible cells accumulated double-membraned autophagic vesicle under electron microscopy and induced GFP-LC3 from diffuse staining to small puncta structure [20]. These data demonstrated that DRAM1 induced autophagy. We transfected HEK293T cells with DRAM1 plasmid and could also observed the turnover of LC3-II from LC3-I as well as the change from the diffuse pattern of GFP-LC3 to puncture structure in the presence of DRAM1, indicating that DRAM1 induced autophagy. We also observed some interesting results upon DRAM1-induced autophagy. For example, in Fig. 1c, Bafilomycin A1 induced accumulation of levels of p62 and LC3-II was blunted by overexpression of DRAM1. Our previous study showed that DRAM1 enhanced autophagic flux through promoting lysosomal acidification [22]. It’s possible that DRAM1 overexpression could partially antagonize the effect of Baf A1 on lysosomes, thus enhance the turnover of autophagosomes and degradation of p62 via lysosome. we also found that DRAM1-induced autophagy involved in the regulation of complexes of autophagosome formation, ULK1 and Atg13. Atg13 localized on the autophagic isolation membrane and is essential for autophagosome formation. ULK1-Atg13 mediated autophagy induction also required mTOR-mediated phosphorylation [23]. The mechanisms of how autophagosome formation complexes is regulated by DRAM1 and whether this regulation is affected by nutrient conditions need further studies.

Our observation that DRAM1 inhibits the phosphorylation of rpS6 suggests that rpS6 might be a signalling target of DRAM1-induced autophagy. RpS6 is targeted by many signals, including growth factors, amino acid, energy, oxygen and osmolarity through various signalling pathways [19]. RpS6 phosphorylation also has multiple physiological roles, such as global protein synthesis, cell proliferation, cell size regulation and glucose homeostasis [19]. As mentioned before, early study showed that insulin and cell swelling promoted amino acid-induced autophagy inhibition, which was associated with the phosphorylation of rpS6 [12]. The relationship between the percentage inhibition of proteolysis and the degree of rpS6 phosphorylation was linear [12]. This study demonstrated that rpS6 phosphorylation is inhibitory for autophagy in hepatocytes, which was consistent with our findings that DRAM1 inhibited rpS6 phosphorylation and induced autophagy. It should be noted that DRAM1 has wide range of tissue expression [25], which suggests that DRAM1 might exert broad functions through inhibiting rpS6 in different tissues. However, whether DRAM1 inhibiting rpS6 is a tissue-specific or a general effect and its role in autophagy regulation in different tissues are still unknown.

Our results demonstrated that DRAM1 exerted an effect on the membrane to regulate the PI3K-Akt pathway. DRAM1 was initially identified as one member of a novel family of proteins including TMEM150 and TMEM77 [25]. The database from uniprot website (https://www.uniprot.org) showed that the TMEM150 family members includes at least TMEM150A, TMEM150B, TMEM150C, DRAM1 and DRAM2. DRAM2 is identified as TMEM77 [26]. These family members are characterized by six-transmembrane domains and share sequence and structural homology among each other [25], whereas their functions are largely unknown. Recently, TMEM150 family members TMEM150A had been shown to regulate the homeostasis of PI(4,5)P_2_ on the plasma membrane in mammalian cells [27]. TMEM150A is a functional homologue of yeast protein Sfk1 in mammalian cells [27]. Another study showed that Sfk1p, a conserved membrane protein in TMEM150/DRAM family, regulate phospholipid asymmetry on the plasma membrane in yeast [28]. These two studies along with the present study emphasized the role of TMEM150 family members on the plasma membrane, which is consistent with the notion that DRAM is an evolutionarily conserved protein from fly to man [29]. The earlier study found that DRAM1 localized on lysosome [20], and subsequent study demonstrated that multiple isoforms of DRAM1 showed broad subcellular localization, including early and late endosomes, autophagosomes, endoplasmic reticulum and peroxisome [21]. It is possible that DRAM1 dynamically shuffled between those membrane-based organelles using membrane trafficking system, especially from plasma membrane to early/late endosome and then to lysosome through the highly dynamic endosome-lysosome network. Our effort to find potential proteins interacted with DRAM1 has no results yet. Identification of the interactors of DRAM1 is a promising way to characterize the role of DRAM1 on the plasma membrane.

Our data that DRAM1 could localize on the membrane and inhibit the activation of IGF-1 receptor upon IGF-1 stimulation propose various possibilities that DRAM1 regulate growth factor receptors. IGF-1 receptor belongs to the tyrosine kinase class of membrane receptors [30]. Many IGF-binding family proteins (IGFBPs) participate in the regulation of IGF biological activity [30]. For example, IGFBP-3 expression was upregulated by transcription factor p53 and was involved in bax-mediated apoptosis. Upregulation of IGFBP-3 could inhibit IGF-stimulated cell cycle and migration, and inhibit apoptosis [31]. DRAM1 is a direct target gene induced by transcription factor p53, it is also possible that DRAM1 acts as an IGFBPs to regulate IGF-1 activity. Autophagy itself includes membrane reorganization and movement. Another interesting possibility is that DRAM1 may accelerate the recycle of growth factor receptors through enhance the membrane dynamic movement, thus weaken the growth factors-induced effects via growth factor receptors. As DRAM1 possesses six-transmembrane domains, it is also possible that DRAM1 act as a receptor on the plasma membrane to direct regulate growth factors. All the assumptions need to be confirmed in the future.

The main methods used in this study to manipulate DRAM1 include plasmid overexpression and siRNA knockdown. Multiple signalling pathway are examined by Western blot analysis in this study. As signalling pathway are vulnerable to external stimuli, serum starvation is introduced to decrease the kinase activity to their basal levels. At this time, stimulation with growth factors will give a more obvious effect. We refer to the method used in Roux’s paper [16]. Cell viability were determined by CCK-8 and colony formation assay. As DRAM1 encodes multiple isoforms [21]. Precise regulation of DRAM1, such as the deletion of DRAM1 by CRISPR/Cas-9, maybe necessary for accurate description of DRAM1’s role.

Our discovery of DRAM1 inhibited rpS6 phosphorylation via the PI3K-Akt-mTORC1-rpS6 pathway provided a mechanistic possibility for understanding the role of DRAM1 in human cancer. Dysregulation of the class I PI3K-Akt-mTOR pathway is the one of most common mechanisms responsible for the development of various types of human cancers [32–34]. DRAM1 has also been reported to be dysregulated in several human cancers [20, 35]. Specifically, DRAM1 mRNA was downregulated in squamous cell carcinoma (SCC) of head and neck tumor samples [20], which highlight the important role of DRAM1 in epithelial cancers. Interestingly, the recent study showed that the PI3K pathway was critical for epidermal homeostasis and the activation of PI3K-dependent signalling pathway, in particular, the PI3K-Akt-mTOR pathway, played a key role in squamous cell carcinoma [36]. Our results that DRAM1 inhibits PI3K-Akt-mTOR pathway might bridges these two isolated phenomena observed in SCC together and provide a new clue for the potential mechanism underlying the tumorigenesis of this type of human cancer. Besides, with the growing number of different inhibitors available for SCC, it would be interesting to test whether DRAM1 could be the promising molecular target of those inhibitors.

As the downstream target of the Akt-mTOR pathway, rpS6 phosphorylation has been viewed as a diagnostic biomarker for the activation of the PI3K-Akt-mTOR pathway [19]. Indeed, highly expressed rpS6 was observed in non-Hodgkin lymphoma [37], and rpS6 activation was responsible for drug resistance in human cancers [38, 39]. While it was until recently that rpS6 had been demonstrated to be the decisive factor for the initiation of pancreatic cancer [40]. Upon implantation of chemical carcinogen DMBA or transgenic expression mutant Kras in pancreatic acinar cells, rpS6^p−/−^ mice (knockin mice lacking all five phosphorylatable sites in rpS6) couldn’t develop pancreatic cancer precursor lesions [40], indicating that rpS6 phosphorylation is essential for tumorigenesis. In addition, they found that rpS6 phosphorylation attenuated Kras-induced DNA damage and p53-mediated tumor suppression [40]. This study connected rpS6 phosphorylation together with p53-induced cell death. Given that DRAM1 is initially identified as the direct target gene of p53 [20, 25] and is essential for p53-induced apoptosis upon DNA damage challenge [20], it is reasonable to speculate that DRAM1 most likely facilities p53-induced apoptosis via inhibiting rpS6 phosphorylation. Furthermore, the pivotal role of rpS6 phosphorylation in tumor formation is consistent with our observation that DRAM1 inhibits rpS6 phosphorylation and act as a tumor suppressor. We believe that rpS6 phosphorylation could serve as the starting point to elucidate the functions of DRAM1 and its role in human cancer.

## Conclusions

This study found that DRAM1-induced autophagy activation was accompanied by the inhibition of rpS6 phosphorylation through inhibiting mTORC1. Further results showed that DRAM1 inhibited the activation of the class I PI3K-Akt pathway. Importantly, DRAM1 regulated the activation of IGF-1 receptor in the plasma membrane. Finally, DRAM1 decreased cell viability upon serum starvation and inhibited rpS6 phosphorylation in human cancer cells. Our finding revealed a novel signalling cascade by which DRAM1 participated in the regulation of autophagy and cell proliferation. Further studies to thoroughly elucidate the role of DRAM1 in tumorigenesis will help to design new strategies for cancer therapy.

## Abbreviations

Baf A1: Bafilomycin A1
DRAM1: Damage-regulated autophagy modulator 1
EGF: Epidermal growth factor
IGF: Insulin-like growth factor
IGF-1R: Insulin-like growth factor 1 receptor
mTOR: mechanistic target of rapamycin
PI3K: Phosphoinositide 3-kinase
rpS6: ribosomal protein S6
